# Case analysis of early-onset Alzheimer's disease associated with TBK1 p.Tyr235Phe gene mutation

**DOI:** 10.3389/fneur.2022.993399

**Published:** 2022-11-03

**Authors:** Pan Li, Yuanyuan Y, Hao Cai, Huihong Zhang, Yuying Zhou

**Affiliations:** ^1^Department of Neurology, Tianjin Huanhu Hospital, Tianjin, China; ^2^Department of Neurology, Tianjin Huanhu Hospital Affiliated to Tianjin Medical University, Tianjin Huanhu Hospital Affiliated to Nankai University, Tianjin University Huanhu Hospital, Tianjin, China; ^3^Tianjin Key Laboratory of Cerebral Vascular and Neurodegenerative Diseases, Tianjin Neurosurgery Institute, Tianjin Huanhu Hospital, Tianjin, China

**Keywords:** TBK1 gene mutation, early-onset Alzheimer's disease, psychobehavioral abnormalities, neuroimaging, biomarkers

## Abstract

TANK1-binding kinase 1 (TBK1) is mainly involved in the regulation of various cellular pathways through the autophagic lysosomal system, and the loss of function or hypofunction caused by TBK1 gene mutation mainly leads to frontotemporal lobar degeneration (FTLD), amyotrophic lateral sclerosis (ALS), and ALS-FTLD. Alzheimer's disease (AD) due to TBK1 gene mutation is extremely rare, and only one case has been reported in China so far. In this report, we described a patient with early-onset AD (EOAD) in whom a new probable pathogenic variant c.704A>T (p.Tyr235Phe) in the TBK1 gene was identified by a whole-genome sequencing analysis. It is suggested that FTLD gene mutation may exist in patients with clinical manifestations of AD.

## Introduction

TANK1-binding kinase 1 (TBK1) is a multifunctional kinase involved in the regulation of multiple cellular pathways, including immune response, inflammation, autophagy, cell proliferation, and insulin signaling (1). It has been demonstrated that TBK1 deficiency will promote serine/threonine-protein kinase 1 (RIPK1)-dependent apoptosis and synergize with genetic risk factors to promote neuroinflammation and lead to the onset of neurodegenerative disorders (2). It has been reported that loss of function (LoF) mutations in the TBK1 gene are the fourth common frontotemporal lobar degeneration (FTLD)-causing gene after repeat expansions in the chromosome 9 open reading frame 72 (C9ORF72), progranulin (GRN), and microtubule-associated protein tau (MAPT), and the second most common ALS-causing gene after C9ORF72 (3). Other rare phenotypes caused by TBK1, such as corticobasal degeneration (CBD) (4) or Alzheimer's disease (AD) (5), have been rarely reported. In this report, we described a case of a TBK1 c.704A>T (p.Tyr235Phe) carrier patient with episodic memory impairment as a prominent manifestation, accompanied by rapid onset of personality changes and behavioral abnormalities. The clinical, neuropsychological, and neuroimaging features and biological markers were consistent with a diagnosis of AD. Therefore, screening for mutations in other dementia-related genes is also needed in clinically diagnosed patients with AD.

## Case report

### Clinical history

The patient is a 53-year-old man and a university professor with a bachelor's degree, who visited our cognitive impairment clinic on 23 November 2020, mainly because of "progressive worsening of memory loss for more than 2 years. The patient was found to have reduced responsiveness during communication, was unable to remember things, and repeatedly asked questions about things he was told by his wife 2 years before the consultation. However, his normal work was not affected. One and a half year ago, the patient felt that his memory loss was deteriorating. He often forgot to give lessons to students and needed to record things to help himself remember. His ability to perform daily activities, such as teaching online, sending emails, managing money, and cooking, has declined. He was irritable and had sleep disturbances, which attracted the attention of his family and sent him to the hospital. He had no significant medical history, denying arterial hypertension, diabetes, coronary heart disease (CHD), cerebrovascular disease, and other chronic illnesses. Also, he had no history of infectious diseases such as hepatitis, tuberculosis, and malaria; history of trauma and blood transfusion; and history of food and drug allergy. According to his family, his uncle suffered from memory loss in his 50 s, which gradually progressed to be unable to recognize his family, accompanied by nocturnal sleep disturbances and abnormal behavioral symptoms, but he did not receive formal treatment until his death in his 60 s. The results of physical examination indicated a temperature of 36.5°C, a pulse of 74 times/min, a breathing rate of 19 times/min, blood pressure (BP) of 128/65 mmHg (1 mmHg = 0.133 kPa), no deformity in the thorax, and clear breath sounds in both lungs, with no dry and wet rales. The heart sounds were strong and rhythmic, and no pathological murmur was heard in the auscultation area of each valve. The abdomen was soft, with no pressure pain, and the liver and spleen were not palpable under the ribs. Both lower limbs were not swollen, and there was no deformity of the spinal limbs. Neurological examination showed no abnormalities except for a decrease in time and place orientation, calculation, and recent memory. The frontal lobe release signs of palm-grasping reflex, rooting reflex, and sucking reflex were all negative.

### Auxiliary examinations

**Figure 1:**
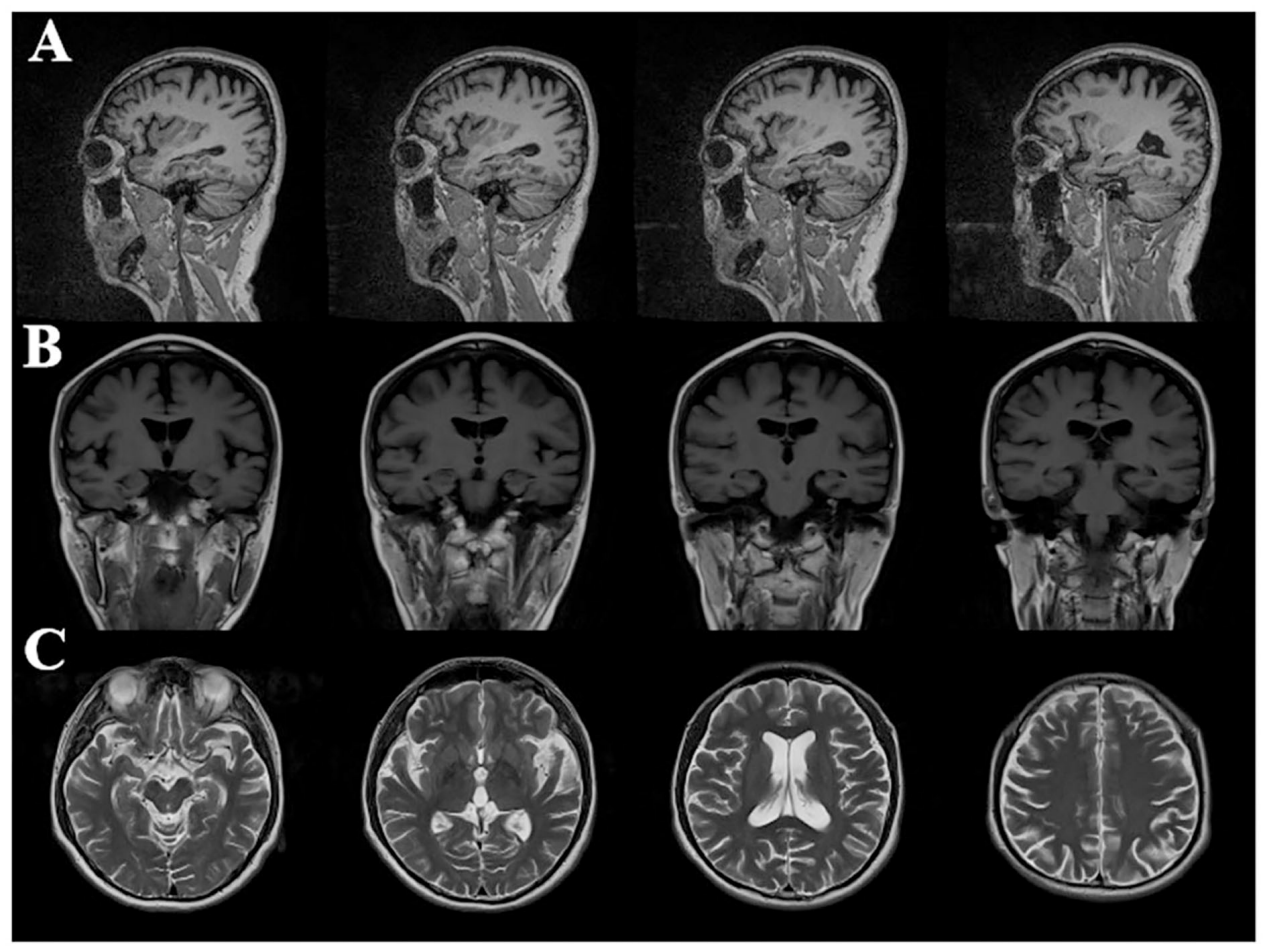
Cranial magnetic resonance imaging in patients with Alzheimer's disease: **(A)** sagittal image of T2 fluid attenuated inversion recovery (T2 FLAIR); **(B)** coronal image of T1-weighted image (T1WI); **(C)** cross-sectional sequences of T2-weighted image (T2WI); imaging results showed atrophy of bilateral temporal parietal lobe and posterior cingulate gyrus, and dilatation of ventricle, cistern, fissure, and sulci.

**Figure 2:**
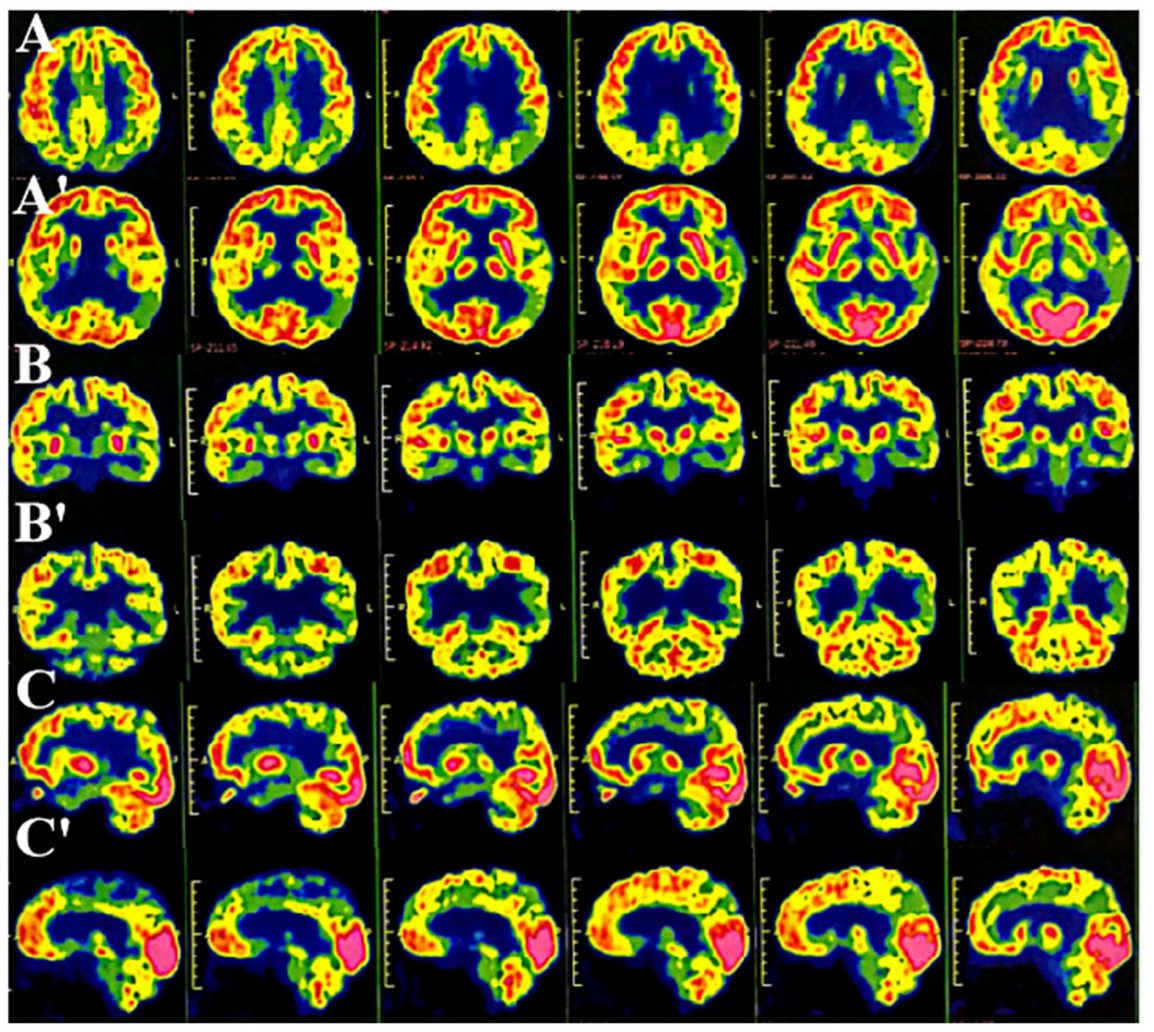
Multimode molecular imaging of 18-fluoro-2-deoxy-D-glucose positron emission tomography (^18^F-FDG PET-CT) showed reduced multiple hypometabolism in the bilateral parietal and temporoparietal junction, bilateral temporal lobes (left), and posterior cingulate gyrus (**AA':** cross-sectional sequence; **BB':** coronal image; **CC':** sagittal image).

The routine blood, coagulation function, serum immune function, liver and kidney function, lipids, blood glucose, high sensitive C-reactive protein, homocysteine, thyroid function, folic acid, vitamin B12, ferritin, immune panel (hepatitis A, hepatitis B, hepatitis C, syphilis, and HIV), urine routine, and stool routine showed no significant abnormalities. The electrocardiogram showed normal sinus rhythm. The neuropsychological assessment showed a multidomain cognitive decline highlighted by delayed recall deficits, along with emotional irritability and sleep disturbances (Table 1). Brain magnetic resonance imaging (MRI) (November 23, 2020) showed atrophy of the bilateral temporoparietal and posterior cingulate gyrus, and widening of the ventricles, cisterna, brain fissures, and brain sulci (Figure 1). Automated brain tissue segmentation was performed by the Dr. Brain tool to extract multiparameter volumetric measurements from different brain regions (https://cloud.drbrain.net, registration number: 20212210359). The artificial intelligence brain structure imaging analysis showed decreased total white matter volume in the whole brain; decreased volume in the bilateral hippocampus, amygdala, left inferior parietal area, and left temporal pole; and thinning of cortical thickness in the bilateral inferior parietal gyrus, left superior parietal gyrus, left superior limbic gyrus, left precuneus, bilateral middle temporal gyrus, left olfactory area cortex, left posterior cingulate gyrus, and bilateral isthmus (Table 2). Multimodal molecular imaging of 18-fluoro-2-deoxy-D-glucose positron emission tomography (^18^F-FDG PET-CT) results suggested the multiple hypometabolism in the bilateral parietal and combined temporoparietal areas, bilateral temporal lobes, and posterior cingulate gyrus (Figure 2). Cerebrospinal fluid (CSF) A*β* and tau were detected by the enzyme-linked immunosorbent assay (ELISA) (6, 7): beta-amyloid (1–42) (A*β*_1 − 42_) was 191.15 pg/ml (normal values >651 pg/ml), beta-amyloid (1–40) (A*β*_1 − 40_) was 7,945.40 pg/ml (normal values >7,000 pg/ml), A*β*_1 − 42_/A*β*_1 − 40_ ratio was 0.024 (normal value > 0.05), total tau protein was 635.89 pg/ml (normal value ≤ 399 pg/ml), and phosphorylated tau181 (p-tau181) protein was 19.8 pg/ml (normal value ≤ 50 pg/ml). Combined with the medical history, clinical symptoms, and relevant examinations, mild cognitive impairment (MCI) due to AD (multidomain amnesia type) was considered according to the revised consensus for MCI diagnosis from the International Working Group (8).

**Table 1:** Neuropsychological scale outcomes for the first visit and follow-up.

**Neuropsychological tests**	**First visit**	**3 months**	**6 months**	**9 months**	**12 months**
MoCA [score/total score (prominent deficit subitems)]	18/30 (delayed recall, visuospatial orientation and executive function)	18/30 (delayed recall, visuospatial orientation and executive function)	17/30 (delayed recall, visuospatial orientation and executive function)	15/30 (delayed recall, visuospatial orientation and executive function)	16/30 (delayed recall, visuospatial orientation and executive function)
MMSE [score/total score (prominent deficit subitems)]	24/30 (delayed recall)	23/30 (delayed recall)	23/30 (delayed recall)	19/30 (delayed recall, orientation, and calculation)	21/30 (delayed recall, orientation, and calculation)
**ADAS-cog (score/total scores)**	Dec-70	Dec-70	14/70	14/70	16/70
Subitems scores:					
Word recall	6	6	6	6	6
Naming	0	0	0	0	0
Commands	0	0	1	1	0
Constructional praxis	1	1	1	1	1
Ideational praxis	1	1	1	1	2
Orientation	1	1	1	1	1
Word recognition	3	3	4	4	6
Remembering test instructions	0	0	0	0	0
Comprehension of spoken language	0	0	0	0	0
Word finding difficulty	0	0	0	0	0
Language	0	0	0	0	0
CDR [score/total score]	0.5/3	0.5/3	0.5/3	3-Jan	0.5/3
Immediate memory [score (abnormal value)]	14 ( ≤ 18)	14 ( ≤ 18)	7 ( ≤ 18)	5 ( ≤ 18)	8 ( ≤ 18)
Delayed recall [score (abnormal value)]	0 ( ≤ 6)	0 ( ≤ 6)	0 ( ≤ 6)	0 ( ≤ 6)	3 ( ≤ 6)
**DST (the longest digit sequence recalled properly)**					
Digits forwards [score/total score]					
Digits backwards [score/total score]	10-Jul	10-Jul	10-Jul	10-Jul	10-Aug
	8-May	8-May	8-Apr	8-Feb	8-Mar
**Trail making test**					
Part A	50 s/0	55 s/0	67 s/0	51 s/0	48 s/0
[time in seconds/errors]					
Part B	123 s/0	127 s/0	101 s/0	118 s/0	116 s/0
[time in seconds/errors]					
ADL [score/total score (prominent deficit subitems)]	24/80 (shopping and financial management)	22/80 (shopping and financial management)	24/80 (hopping, financial management and complicated housework)	25/80 (shopping, financial management and complicated housework)	25/80 (shopping, financial management and complicated housework)
NPI [score/total score (prominent deficit subitems)]	4/122 (sleep disturbance and irritability)	2/122 (sleep disturbance and irritability)	2/122 (sleep disturbance and irritability)	3/122 (Stubborn and irritability)	4/122 (Stubborn and irritability)
HAMD-17 [score/total score (prominent deficit subitems)]	9/55 (Sleep disturbances, reduced ability to work)	9/55(Sleep disturbances, reduced ability to work)	8/55 (Sleep disturbances, reduced ability to work)	9/55 (Sleep disturbances, reduced ability to work)	8/55 (Sleep disturbances, reduced ability to work)

MMSE, Mini-Mental State Examination; MoCA, Montreal Cognitive Assessment; ADAS-cog, Alzheimer's Disease Assessment Scale-Cognitive section; CDR, Clinical Dementia Rating; DST, Digital Span Test; ADL, Activity of Daily Life; NPI, Neuropsychiatric Inventory Questionnaire; HADM-17, The 17-items Hamilton Depression Rating Scale.

**Table 2:** The structural MRI features based on artificial intelligence analysis.

	**Structure**	**Volume (cm^3^)**	**Ratio to intracranial volume (%)**	**Normal range (%)**
The whole brain information	Gray matter	574.93	39.45%	35.94–47.91%
	White matter	460.03	31.57% ↓	31.62–41.96%
	Total cerebrospinal fluid	422.27	28.98%	11.78–30.24%
	Total intracranial space	1457.23	–	–
Hippocampus	Left hippocampus	1.93	0.13% ↓	0.16–0.24%
	Right hippocampus	2.48	0.17% ↓	0.18–0.27%
	Left Parahippocampus Gyrus	2.15	0.15%	0.15–0.22%
	Right Parahippocampus Gyrus	2.25	0.15%	0.15–0.22%
Amygdala	Left amygdala	0.58	0.04% ↓	0.05–0.07%
	Right amygdala	0.63	0.04% ↓	0.05–0.07%
	**Structure**	**Cortical thickness (mm)**	**Normal range (mm)**	
Cortical structure	Left inferior parietal gyrus	1.9 ↓	2.2–2.6	
	Left superior parietal gyrus	1.88 ↓	1.9–2.4	
	Left supramarginal gyrus	2.18 ↓	2.2–2.7	
	Left precuneus	1.99 ↓	2.1–2.6	
	Left middle temporal gyrus	2.42 ↓	2.5–3.1	
	Left entorhinal cortex	2.87 ↓	2.9–4.0	
	Left posterior cingutate	2.13 ↓	2.2–2.8	
	Left isthmus	1.98 ↓	2.0–2.7	
	Right inferior parietal gyrus	2.11 ↓	2.2–2.6	
	Right middle temporal gyrus	2.58 ↓	2.6–3.1	
	Right isthmus	1.95 ↓	2.0–2.7	

### Treatment and follow-up

**Figure 3:**
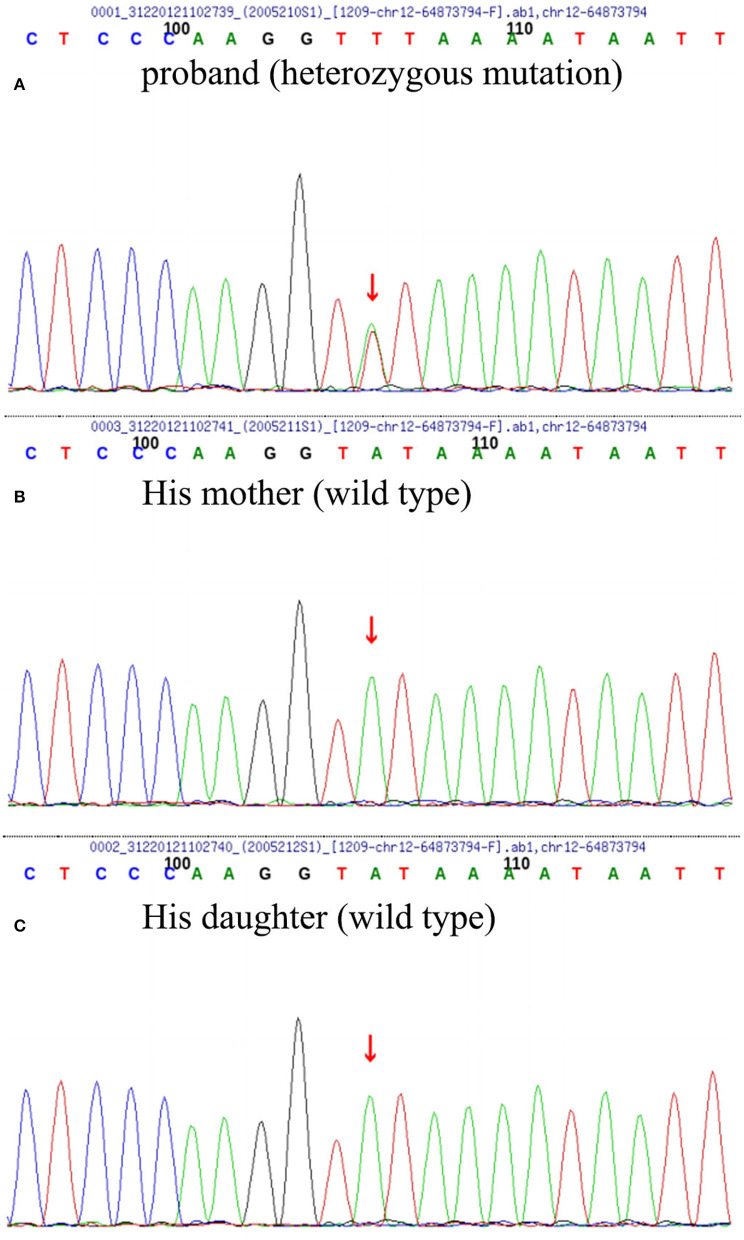
Whole-genome sequencing (WGS) and repeated primer PCR detection results of the patient **(A)**, his mother **(B)**, and daughter **(C)** the patient carried heterozygous missense mutation of *TBK1* c. 704A>T (p. Tyr235phe); however, his daughter and mother were wild type.

Butylphthalide (0.6 g/day) was given orally. At 3 months, the patient's cognitive function and neuropsychiatric symptoms remained stable. At 6 months, immediate memory function began to be impaired and the activity of daily living function has worsened. At 9 months, the patient showed significant personality changes and behavioral abnormalities, manifested by stubbornness, repeated excessive shopping, confabulating, irritability, and disinhibition. At the same time, cognitive function (orientation and calculation) has declined more than before (Table 1). Molecular genetic screening was performed by whole-genome sequencing and repeat primer PCR: the patient carried a TBK1 c.704 A>T (p.Tyr235Phe) heterozygous mutation (Figure 3) with an APOE genotype of ε2/ε4. Further validation of the locus by Sanger sequencing was performed in the proband's family, and both his mother and daughter were wild type. Accordingly, the medication was adjusted to memantine and donepezil.

## Discussion

Alzheimer's disease is a chronic progressive neurodegenerative disorder, and there is still a lack of effective and accurate diagnostic methods for early identification. AD is highly heterogeneous in terms of clinical manifestation and neuropathology, and the prominent manifestation of “episodic memory impairment” as a diagnosis of AD lacks high sensitivity and specificity for early diagnosis (9). On the contrary, even in the pathologically confirmed population with AD, FTLD-like phenotypes such as “neuropsychiatric abnormalities, personality and language disorders” and movement disorder phenotypes such as “myoclonus, epilepsy and spastic paraplegia” can also be manifested (10). Neuropathology remains the gold standard for AD diagnosis. In gross pathology, AD presents as diffuse brain atrophy, with typical AD dominated by hippocampal and medial temporal lobe atrophy, posterior variant AD by the temporal occipital lobe and posterior cingulate gyrus atrophy, frontal variant of AD (fvAD) by bilateral frontal lobe atrophy, and logopenic variant AD by left parietal–temporal lobe atrophy; however, these macroscopic features are a lack of specificity for AD diagnosis. In the microscopic pathology, in addition to typical pathological changes of extracellular amyloid plaque deposition and intracellular neurofibrillary tangles, AD also includes eosinophilic inclusions, hippocampal granular vacuolar degeneration, activated microglia, reactive astrocytes, amyloid vascular disease, and other pathological changes (9). Neurodegenerative diseases are characterized by the accumulation and deposition of misfolded proteins in the brain, most notably A*β*, tau, alpha-synuclein, and TAR DNA-binding protein 43 (TDP-43) (11), and may also be referred to as proteinopathies. Although the characteristic proteins differ across the disease spectrum, a growing number of studies confirm the prevalence of co-pathologies (12). Among the patients clinically diagnosed with AD, autopsy results showed that only 17.3–26.3% had AD pathology alone, 28.3–41.6% had Lewy body dementia, 13.9–49.2% had vascular dementia, and even 12.6% had no AD pathological changes (11, 13, 14). Conversely, some pathological alterations, such as tau protein and TDP-43 caused by FTLD-related pathogenic gene mutations of GRN, C9ORF72, and MAPT, can also be clinically manifested as a typical AD phenotype (15–17). The frequent occurrence of co-pathology will contribute to the heterogeneity of clinical symptoms, making the differential diagnosis challenging. In this study, we have identified a novel heterozygous mutation in FTLD- and ASL-related pathogenic genes in a patient with AD phenotype, Therefore, screening for mutations in TBK1 might be advisable in clinically diagnosed patients with AD.

An LoF mutation in the TBK1 gene was first identified in 2015 in a cohort study of patients with ALS, resulting in the degradation of mutant transcripts and reduced TBK1 protein. Subsequently, the same mutations were identified in the FTLD population (18). TBK1 LoF mutations are the third most frequent cause of clinical FTLD in the Belgian clinically based patient cohort, after C9ORF72 and GRN, and the second most common cause of clinical ALS after C9ORF72 (19). These findings reinforce that FTLD and ALS belong to the same disease continuum. TBK1 protein has four functional domains, including a serine/threonine kinase (S/TK) domain, which phosphorylates TBK1 substrates, a ubiquitin-like structural domain, and two coiled-coiled domains (CCD1 and CCD2). The CCD2 domain binds to optineurin and p62, the two key proteins involved in the autophagic pathway, and promotes substrate phosphorylation, thereby participating in neurodegenerative changes in ALS and FTLD (20). The correlation between the location of TBK1 LoF mutations and clinical phenotypes is still controversial. It has been reported that in patients with ALS and related dementia, TBK1 missense mutations are mostly located near the CCD2 domain and will affect its combination ability to optic nerve protein (18). Some studies have also proposed that missense mutations associated with FTLD and FTLD-ALS are always located in the S/TK or CDD2 domains (20). Genetic screening in this patient suggested a TBK1 c.704A>T (p.Tyr235Phe) heterozygous mutation, located in the S/TK structural domain, which is the first reported locus.

TBK1 mutation carriers mainly present with prominent psychological and behavioral abnormalities, such as apathy and disinhibition, and can also be accompanied by memory loss in the early stage of the disease. Some patients exhibit significant upper motor neuron symptoms and progressive medullary paralysis. More than half of TBK1 LoF mutation or missense mutation carriers are clinically diagnosed with pure ALS, FTLD, and ALS-FTLD (21), and rarely patients present with other neurodegenerative disorders such as CBD (4), progressive supranuclear palsy (PSP) (21), progressive cerebellar ataxia (PCA) (21), and AD (5). A systematic screening of the coding sequence of TBK1 in a large cohort of 1,253 patients from eight European countries to investigate the frequency of TBK1 LoF mutations in the population with AD identified only 1 LoF mutation (p.Thr79del) in a patient clinically diagnosed with AD in a positive familial ALS cohort. It was the only reported case abroad at present (5). The patient was clinically diagnosed with sporadic EOAD at the age of 62 years, with the onset of first symptoms at 59 years old. The initial symptoms were visuospatial disorientation and recent memory deficits, and imaging suggested medial temporal lobe atrophy. The progressive cognitive decline was consistent with the characteristics of AD. With disease progression, frontal features became apparent, followed by bilateral parkinsonism at the late stages (5). A domestic research of gene mutations in a Han Chinese AD cohort showed a novel, heterozygous missense mutation at the TBK1 p.D534H locus with a typical AD phenotype of memory loss and disorientation (22). Although the common variant associated with reduced TBK1 expression may be more enriched in patients with EOAD than in controls, this requires further confirmation given the lack of association in late-onset AD, and further investigation of common variants affecting TBK1 expression is warranted (5).

In the present report, the patient was diagnosed with sporadic EOAD at 53 years old and had the onset of first symptoms at 51 years old. His clinical evaluation was considered to be in line with AD-type dementia due to the following supporting evidence: (1) recent memory decline was its early prominent manifestation, combined with visuospatial disorientation and other cognitive subdomains dysfunction; (2) multimodal imaging analysis of brain structure and PET-CT molecular imaging results suggested bilateral temporal lobe, parietal lobe and combined temporoparietal regions atrophy and hypometabolism, and multiple metabolic restrictive hypometabolism in the posterior cingulate gyrus; and (3) the decrease in A*β*_1 − 42_ and A*β*_1 − 42_/A*β*_1 − 40_ ratio and the increase in total tau protein found in CSF. It is consistent with the diagnosis of typical AD according to clinical, neuropsychological, neuroimaging features and biological markers. However, during the follow-up, the patient soon developed behavioral abnormalities, such as disinhibition and stereotyped compulsive behavior, which could not exclude the modifying effect of TBK1 heterozygous mutation on the disease process and required to be tracked continuously. Actually, according to the new diagnostic criteria proposed by Ossenkoppele (23), the patient is clinically most reminiscent of fvAD. FvAD is a variant form of AD characterized by a milder and more restricted behavioral profile than in behavioral variant frontotemporal dementia, as well as the co-occurrence of memory dysfunction and high APOE ε4 prevalence; however, it shares most pathophysiological features with typical AD. It is worthy of further investigation that A*β*_1 − 42_/A*β*_1 − 40_ ratio and total tau protein level were altered in the CSF, whereas there was no change in the level of p-tau181. Numerous clinical studies have been presented that p-tau181 concentration is a promising new biomarker candidate for AD diagnosis and prognosis, and as the earliest reactive protein to A*β* toxicity, the p-tau181 level can accurately predict the state of A*β* deposition in the brain (24). However, an analysis obtained from the AD Neuroimaging Initiative (ADNI) suggested that nearly 20% of the study population showed A*β* positive and p-tau181 negative phenotype (25). It is possible that there are multiple pathologies, other than *β*-amyloid plaques and neurofibrillary tangles, that synergistically contribute to brain damage in the patient (26).

In conclusion, our data report a case with a typical AD phenotype carrying TBK1 LoF variant with a biomarker-supported diagnosis of AD, which is the second case reported in China, and the phenotypic characteristics of the TBK1 c.704A>T (p.Tyr235Phe) heterozygous mutation are reported in the first case. Considering the development of frontal features in the course of the disease, this does not entirely exclude the possibility that this patient had co-existed FTLD with atypical clinical presentation due to early symptoms compatible with AD. This is consistent with the previous TBK1 LoF variation found in FTLD/ALS patients with a preliminary clinical diagnosis of AD. This study also suggests that FTLD gene mutations may also occur in clinically diagnosed patients with AD; hence, screening for mutations in other dementia genes in clinically diagnosed patients with AD may be desirable. The onset of the clinical pictures and the natural history is highly different in neurodegenerative diseases caused by TBK1 gene mutation. It is difficult to establish genotype–phenotype correlations because of the molecular complexity of TBK1 and pathological heterogeneity in carriers of TBK1 mutations. Thereby, further studies are needed to better understand the pathophysiology of TBK1, to provide comprehensive genetic counseling in affected families, and to improve prevention strategies as well as treatments.

## Data availability statement

The datasets presented in this article are not readily available because of ethical and privacy restrictions. Requests to access the datasets should be directed to the corresponding author.

## Ethics statement

The studies involving human participants were reviewed and approved by Tianjin Human Trial Committee and approved by Ethics Committee of Tianjin Huanhu Hospital. The patients/participants provided their written informed consent to participate in this study. Written informed consent was obtained from the individual(s) for the publication of any potentially identifiable images or data included in this article.

## Author contributions

PL wrote the original manuscript and made modifications. HC and HZ performed the data collection. YZ and PL participated in clinical diagnosis. YY and HZ were responsible for patient care and scale assessment. All authors examined the results and authorized the final version of the manuscript.

## Funding

This study was supported by Tianjin Municipal Health Commission Project (TJWJ2021MS029) and Tianjin Key Medical Discipline (Specialty) Construction Project (No. TJYXZDXK-052B). Both funds are chaired by PL.

## Conflict of interest

The authors declare that the research was conducted in the absence of any commercial or financial relationships that could be construed as a potential conflict of interest.

## Publisher's note

All claims expressed in this article are solely those of the authors and do not necessarily represent those of their affiliated organizations, or those of the publisher, the editors and the reviewers. Any product that may be evaluated in this article, or claim that may be made by its manufacturer, is not guaranteed or endorsed by the publisher.
