# Supplementary material for: Case analysis of early-onset Alzheimer's disease associated with TBK1 p.Tyr235Phe gene mutation
Source: Front Neurol. 2022 Nov 3;13:993399. doi: 10.3389/fneur.2022.993399 (PMC9671219; doi:10.3389/fneur.2022.993399)
Supplement: Supplementary file 1 [file Data_Sheet_1.PDF]

立即回忆
Immediate memory：

指导语：
Instructions：

下面我给您念一些物体的词，我念完以后，请您把记住的词语告诉我，可以不按顺序说，记得什么就说什么。然后我会再给您念几遍，请您把每次记住的词都告诉我，包括前一次已经说过的词，看您最后能记住多少，好吗？

Now I will read you some words about objects. When I finish reading, please tell me the words you remember. You can say them out of order, just say what you remember. Then I will read it to you several times., please tell me the words you remember each time, including the words you said the last time, and record how many words you can remember in the end, okay?

检查者以每个词 1 秒的速度清楚的读出，用 1, 2, 3 等阿拉伯数字进行记录，同时记录插入数和重复数。同 1 物品两次以上插入算为 1 个插入，每次重复均算为 1 次重复，但对插入物品的重复不计入重复。每次两分钟回忆时间。

The examiner read each word clearly at a rate of 1 second, using 1, 2, and 3 Arabic numerals, as well as the number of insertions and repeats. More than two insertions of the same object are counted as one insertion, and each repetition is counted as one repetition, but the repetition of the inserted object is not counted as a repetition. Two minutes for recall at a time.

如果近 3 个月受试者曾做过这个检查，记录检查日期。
If the subject has had this test in the last 3 months, pleases record the date.

项目　objects	第一遍 the first time	第二遍 the second time	第三遍 the third time
胳膊　arm			
猫　cat			
斧子　hatchet/axe			
床　bed			
飞机　airplane			
耳朵　ear			
狗　dog			
锤子　hammer			
椅子　chair			
轿车　car			
眼睛　eye			
马　horse			
刀子　knife			
钟　clock			
自行车 bicycle			
正确个数<br>The number of correct recall	□□	□□	□□
插入单词记录<br>Insert word record	□□	□□	□□
插入数<br>The number of insertion	□□	□□	□□
重复数<br>The number of repetition	□□	□□	□□
正确的个数<br>The number of correct	3 遍正确回忆总数<br>The total number of correct recall in three times	□□　（异常参考值≦18）<br>□□　（Abnormal value≦18）	
插入数<br>The number of insertion	3 遍插入总数<br>The total number of insertion in three times	□□<br>□□	
重复数<br>The number of repetition	3 遍重复回忆总数<br>The total number of repetition in three times	□□<br>□□	
记忆策略（根据物体分类记忆）<br>Memory strategies (Remember according to object categorization)		☐有　☐无<br>☐Yes　☐No	

延迟回忆
Delayed recall

指导语：
Instructions：

还记得我刚才给您念了好几遍的那些词吗？请您尽可能多的回忆出来。（2 分钟回忆时间，用 1，2，3 等阿拉伯数字进行记录，同时记录插入数和重复数。同 1 物品多次插入算为 1 个插入，每次重复均算为 1 次重复，但对插入物品的重复不计入重复。）

Do you remember the words I have just read to you several times? Please remember as many as you can. (Two minutes of recall time, using 1, 2, and 3 Arabic numerals, as well as the number of insertions and repeats. Multiple insertions of the same object are counted as one insertion, and each repetition is counted as one repetition, but the repetition of the inserted object is not counted as a repetition.)

胳膊 arm		耳朵 ear		眼睛 eye	
猫 cat		狗 dog		马 horse	
斧子 hatchet/axe		锤子 hammer		刀子 knife	
床 bed		椅子 chair		钟 clock	
飞机 airplane		轿车 car		自行车 bicycle	
正确回忆个数<br>The number of correct recall	☐☐ （异常参考值≦6）<br>☐☐ （Abnormal value≦6）				
插入单词记录<br>Insert word record	______________________________				
插入数<br>The number of insertion	☐☐<br>☐☐				
重复数<br>The number of repetition	☐☐<br>☐☐				
记忆策略（根据物体分类记忆）<br>Memory strategies (Remember according to object categorization)			☐有 ☐无<br>☐Yes ☐No		
